# Temperature-dependent survival of *Mycoplasma anserisalpingitidis* in water: implications for biosecurity and transmission in waterfowl farming

**DOI:** 10.1186/s12917-025-05005-2

**Published:** 2025-10-13

**Authors:** Anna Sawicka-Durkalec, Zsuzsa Kreizinger, Dénes Grózner, Olimpia Kursa, Grzegorz Tomczyk, Miklós Gyuranecz

**Affiliations:** 1 National Veterinary Research Institute, Aleja Partyzantów 57, Puławy, 24-100 Poland; 2 HUN-REN Veterinary Medical Research Institute, Hungária körút 21, Budapest, 1143 Hungary; 3 National Laboratory of Infectious Animal Diseases, Antimicrobial Resistance, Veterinary Public Health and Food Chain Safety, István utca 2, Budapest, 1078 Hungary

**Keywords:** Avian mycoplasma, *Mycoplasma anserisalpingitidis*, Pathogen survival, Temperature adaptation, Waterfowl

## Abstract

**Background:**

*Mycoplasma anserisalpingitidis* is an emerging waterfowl pathogen associated with reproductive tract infections, embryo mortality, and reduced egg production. While direct and vertical transmission routes have been described, its environmental persistence remains poorly understood. In waterfowl production systems, open water sources can be used for drinking and bathing, potentially facilitating indirect transmission. Prolonged survival in such environments may extend the period during which birds are exposed to the pathogen. Understanding the ability of *M. anserisalpingitidis* to survive outside the host, particularly under environmental stress, is essential for assessing transmission risks.

**Results:**

This study evaluated the survival of two *M. anserisalpingitidis* strains in water at environmental temperatures of 0 °C, 4 °C, and 22 °C. To our knowledge, this is the first study to compare environmental survival between two strains of an avian mycoplasma species. Survival was found to be both temperature-dependent and strain-specific. Strain A remained viable for up to 12 days at 0 °C and 8 days at 4 °C, while strain B survived for the entire 28-day experiment at both temperatures. At 22 °C, strain A lost viability within 24 h, while strain B persisted for 3 days. qPCR analysis of DNA concentrations confirmed these survival patterns, indicating better recovery of strain B under low-temperature conditions.

**Conclusions:**

These findings demonstrate the capacity of *M. anserisalpingitidis* to persist in cold water and highlight the potential role of water sources as environmental reservoirs contributing to indirect transmission in waterfowl farming. The results emphasize the importance of considering water systems as potential environmental reservoirs when designing biosecurity and disease control strategies.

**Supplementary Information:**

The online version contains supplementary material available at 10.1186/s12917-025-05005-2.

## Background

*Mycoplasma anserisalpingitidis* is an important and emerging pathogen in waterfowl, particularly in geese where it causes reproductive tract inflammation, reduced egg production, and embryo mortality, leading to significant economic losses in commercial flocks [1]. Transmission occurs both horizontally through direct contact between birds and vertically via infected eggs [2]. Phylogenetic analyses of isolates from wild geese suggest the potential for long-distance dissemination [3], raising concerns over its environmental persistence and confirming the role of wild birds in pathogen transmission into farming systems. Although vertical and direct horizontal transmission of *M. anserisalpingitidis* have been described, indirect environmental transmission and survival outside the host have not yet been investigated. Little is known about its ability to survive outside the host. For other avian mycoplasmas, such as *M. synoviae* and *M. gallisepticum*, environmental survival and indirect transmission routes have been well documented [4–6]. Among avian mycoplasmas, environmental transmission has been experimentally confirmed for *M. synoviae*, which was shown to cause delayed infection in chicks after exposure to contaminated environments [7]. Additionally, Marois et al. [6] demonstrated that water and feed are among the most effective sample types for environmental detection of *M. gallisepticum* on poultry farms. These findings support the hypothesis that water may serve as an environmental reservoir contributing to the transmission of avian mycoplasmas. However, no studies have evaluated the environmental survival of *M. anserisalpingitidis* in water to date.

This aspect is particularly relevant in waterfowl production systems, where birds frequently contaminate and interact with water used not only for drinking but also for bathing. Although general recommendations regarding water quality have been issued by the World Organisation for Animal Health (WOAH) and the European Food Safety Authority (EFSA), current legal regulations, such as Directive 98/83/EC, primarily cover drinking water intended for human consumption. While in practice these standards are often applied to water used in animal husbandry, including avian species, there are no dedicated microbiological criteria or systematic monitoring requirements tailored specifically to poultry production. This regulatory ambiguity may lead to an underestimation of the risks related to environmental exposure and pathogen transmission in such settings [8, 9]. This is especially concerning for waterfowl, which spend a considerable amount of time in direct contact with open water systems that can serve as both a source and reservoir of infectious agents [10].

Environmental factors such as temperature and nutrient availability are key determinants of bacterial survival. Among abiotic stressors, temperature is one of the most critical factors influencing microbial physiology [11]. Interestingly, the optimal temperature for bacterial survival may be lower than the optimal temperature for growth [12]. Exposure to suboptimal temperatures can lead to structural changes in bacterial proteins, which in turn impair the uptake of essential nutrients required for cell viability [13]. Some bacteria can develop protective adaptation mechanisms in response to environmental stress, including cold and nutrient limitation [14, 15]. These mechanisms may involve structural or metabolic changes that increase persistence under suboptimal conditions. In mycoplasmas, which have minimal genomes and limited metabolic capacity, such adaptations may be particularly important for maintaining viability outside the host [16, 17].

The aim of this study was to evaluate the survival ability of two *M. anserisalpingitidis* strains with different origins under varying temperature conditions in water. Their survival was assessed at three temperatures: 0 °C, 4 °C, and 22 °C, in order to simulate natural environmental conditions. Understanding the environmental resilience of this pathogen is essential for developing effective biosecurity measures and minimizing the risk of environmental transmission in waterfowl farming. Such data are critical for assessing the potential for indirect transmission routes and for guiding targeted control strategies.

## Materials and methods

### Strains and culture preparation

Two strains of *Mycoplasma anserisalpingitidis* were used in this study. Both strains were isolated from cloacal swabs. Strain A originated from a white-fronted goose (*Anser albifrons*) that was legally shot during a regular hunt, and the sample was collected postmortem. Strain B was obtained from a farmed goose (*Anser anser domesticus*) during routine veterinary diagnostics, with the owner’s informed consent. Both isolates were obtained in Poland and stored in the institutional strain collection prior to this study. The strains had previously been purified to ensure homogeneity before further testing. Briefly, they were inoculated using the reduction streaking method [18] onto agar media (MolliScience Kft., Biatorbágy, Hungary) for mycoplasma cultivation and incubated at 37 °C in 5% CO_2_ atmosphere to promote colony growth. Subsequently, one well-developed colony from each strain was selected and transferred to liquid culture media for further propagation. In the next stage, the obtained strains were tested for contamination with other mycoplasma species (*M. anatis*, *M. anseris*, *M. cloacale* [19] and *Acholeplasma laidlawii* [20]) to confirm strain purity before experimental inoculation. For this purpose, genetic material was isolated from the strains and analyzed using real-time PCR and conventional PCR methods targeting the mentioned pathogens. The number of color-changing units (CCU/mL) for each sample was determined at 37 °C in 96-well microtiter plates using the broth microdilution method [21]. This involved performing a series of 10-fold dilutions of the *Mycoplasma* suspension in triplicate. The plates were monitored daily, and the final results were read after 7 days. The highest dilution that exhibited a color change was considered to contain 10⁰ CCU/mL of bacteria. On the day the experiment began, both strains were inoculated onto liquid and solid media to confirm their viability.

### Water

The water used in the experiment was commercially purchased drinking water intended for human consumption. It was tested by the manufacturer for quality parameters, including pH and mineral content, to ensure it met safety standards. Prior to use, the water was stored in its original sealed bottle at room temperature.

### Inoculation and incubation of samples

Appropriate volumes of water were prepared to conduct the experiment and then inoculated with strains A and B to achieve a final concentration of 10⁵ CCU/mL. After inoculation, the water was carefully mixed and divided into individual samples with a volume of 2 mL, which were placed at designated incubation temperatures. Samples were incubated at 0 °C, 4 °C, and 22 °C to simulate environmental temperatures that may be encountered in natural or farming conditions. To determine and compare the survival of both strains, the assay was performed in triplicate, with each run following the same experimental design. To maintain the target temperature of 0 °C, samples were incubated in a refrigerated container filled with ice, and the temperature was monitored with a separate calibrated thermometer (0.1–0.5 °C). Ice was replenished daily to ensure consistent temperature. Samples incubated at approximately 4 °C were stored in a refrigerator, while those intended for survival studies at 22 °C were kept in a temperature-controlled room.

### Evaluation of bacterial survival

To assess viability, 200 µL of each *Mycoplasma*-inoculated water sample was inoculated into 2 mL of General Mycoplasma liquid medium (MolliScience Kft., Biatorbágy, Hungary). This was performed on days 1, 2, 3, 5, 8, 12, 17, 23, and 28 of the experiment. All broth cultures were incubated at 37 °C and checked daily for color change. As soon as a change was observed, each culture was plated onto agar plates General Mycoplasma solid medium (MolliScience Kft., Biatorbágy, Hungary). Drops of the cultures were applied to the agar and incubated at 37 °C with 5% CO_2_ for 14 days, or until characteristic colonies developed. To confirm negative results, all samples without visible color change were additionally plated on agar medium and incubated for 14 days under identical conditions. The plates were checked daily for the presence of characteristic colonies.

### DNA extraction and quantitative PCR (qPCR) analysis

DNA was extracted from 200 µL of broth culture using a QIAamp DNA Mini Kit (Qiagen, Hilden, Germany), following the manufacturer’s instructions. The extracted DNA was immediately frozen and stored at − 20 °C for further analysis. The same extraction protocol was applied to *Mycoplasma*-inoculated water samples in which the strain had been incubated. Quantitative polymerase chain reaction (qPCR) was used to detect *M. anserisalpingitidis*, with primers and a probe targeting the alpha subunit (*rpoA*) as described by Bekő et al. [22]. The reaction was performed using a QuantiTect Probe PCR Kit (Qiagen) with a total volume of 25 µL, consisting of 12.5 µL of master mix, 1.3 µL of each 10 µM primer, 0.5 µL of probe, 7.4 µL of distilled water, and 2 µL of DNA. Amplification was carried out in an ABI 7500 Real-Time PCR System (Applied Biosystems, Thermo Fisher Scientific, Waltham, MA, USA) with initial denaturation at 95 °C for 10 min, followed by 40 cycles of 95 °C for 15 s and 58 °C for 1 min.

### Statistical analysis

Survival of *M. anserisalpingitidis* was assessed based on culture method results. The probability of survival over time was analysed using Kaplan–Meier survival curves, fitted with the ‘survfit’ function from the *survival* package [23]. The three replicates per condition were treated as independent observations and analyzed individually. Each replicate was assigned a binary outcome (positive or negative) based on culture results at each time point, and these values were then used directly in the Kaplan–Meier survival analysis. Survival curves were generated separately for each strain (A and B) and incubation temperature (0 °C, 4 °C, 22 °C). Differences in survival between groups were assessed using the log-rank test (‘survdiff’ function), with the significance level set at *P* < 0.05. Visualisation of the survival curves was performed using the autoplot() function from the *ggfortify* package [24]. DNA concentrations obtained by qPCR in broth and water samples were visualised using the *ggpubr* package [25] as dot whisker plots showing median values and interquartile ranges. Statistical differences in DNA concentration between strains were evaluated using t-test at each time point and within each temperature group using ‘stat_compare_means’ function implemented in the *ggpubr* package. Differences were considered statistically significant at P < 0.05. All statistical analyses were performed using R version 4.4.3 [26] and RStudio version 2024.12.1 + 563 [27].

## Results

**Fig. 1:**
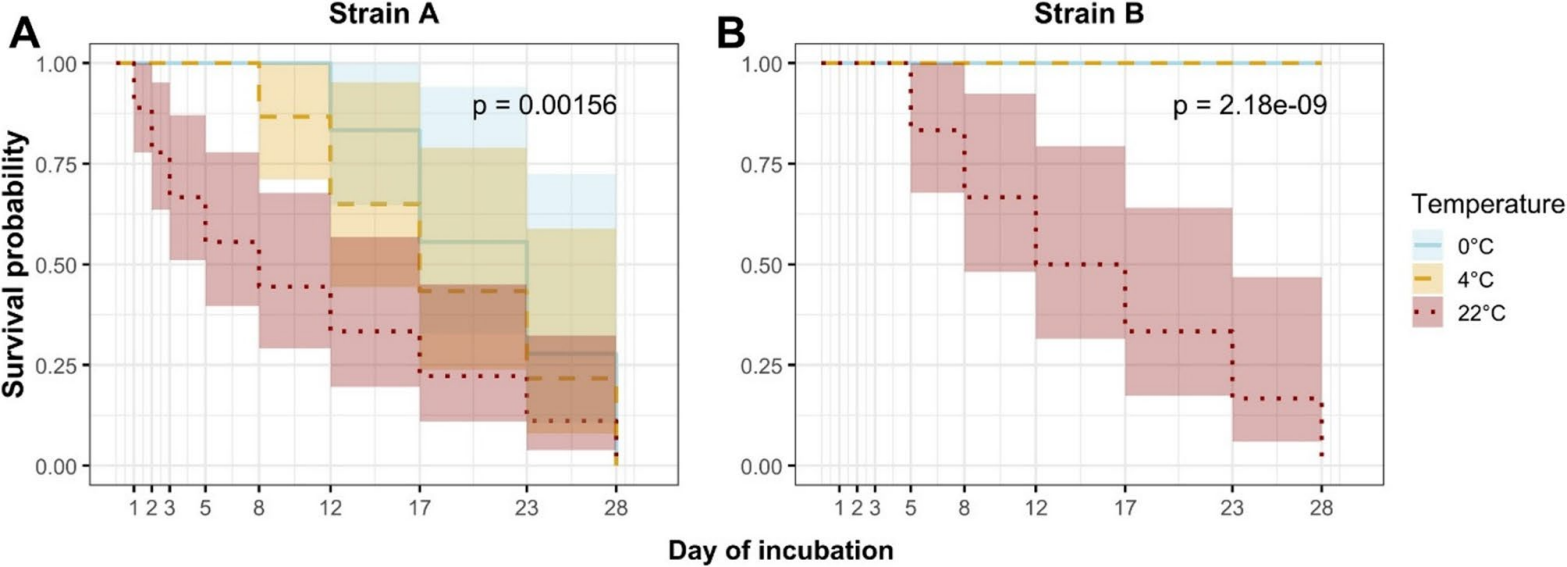
Kaplan-Meier survival curves of *M. anserisalpingitidis* strains **A** and **B** incubated at 0 °C, 4 °C, and 22 °C. Survival was determined based on culture results over a 28-day incubation period. Curves represent the proportion of positive samples over time, with shaded areas indicating 95% confidence intervals. Statistical differences between temperature groups within each strain were assessed using the log-rank test, with the significance level set at *P* < 0.05

The viability of *M. anserisalpingitidis* strains A and B was evaluated over a 28-day period at three incubation temperatures: 0 °C, 4 °C, and 22 °C. Culture-based assays revealed distinct survival patterns between the two strains (Fig. 1).

**Fig. 2:**
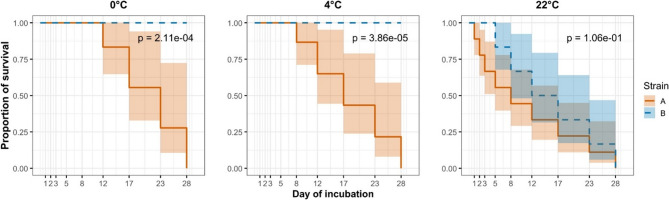
Kaplan-Meier survival curves of *M. anserisalpingitidis* by strain (A and B) incubated at 0 °C, 4 °C, and 22 °C. Survival was determined based on culture results over a 28-day period. Curves represent the proportion of positive samples over time, with shaded areas indicating 95% confidence intervals. Statistical differences between strains within each temperature condition were assessed using the log-rank test, with the significance level set at *P* < 0.05

Strain A exhibited the shortest survival times across all tested conditions. At 0 °C, it remained viable until day 12; at 4 °C, viability persisted until day 8; and at 22 °C, strain A lost viability after just 1 day of incubation. In contrast, strain B demonstrated greater environmental resilience. It survived the full 28 days at both 0 °C and 4 °C, and remained viable until day 3 at 22 °C. Our findings indicate a pronounced difference in temperature sensitivity between the two strains. Notably, even the moderate difference between 0 °C and 4 °C led to a statistically significant reduction in viability for strain A (Fig. 2). At 22 °C, strain A lost viability within the first 24 h, whereas strain B remained viable until day 3 (loss of viability occurred between days 3 and 5). However, the difference in survival between the strains was not statistically significant (*P* = 0.106; Fig. 2).

**Fig. 3:**
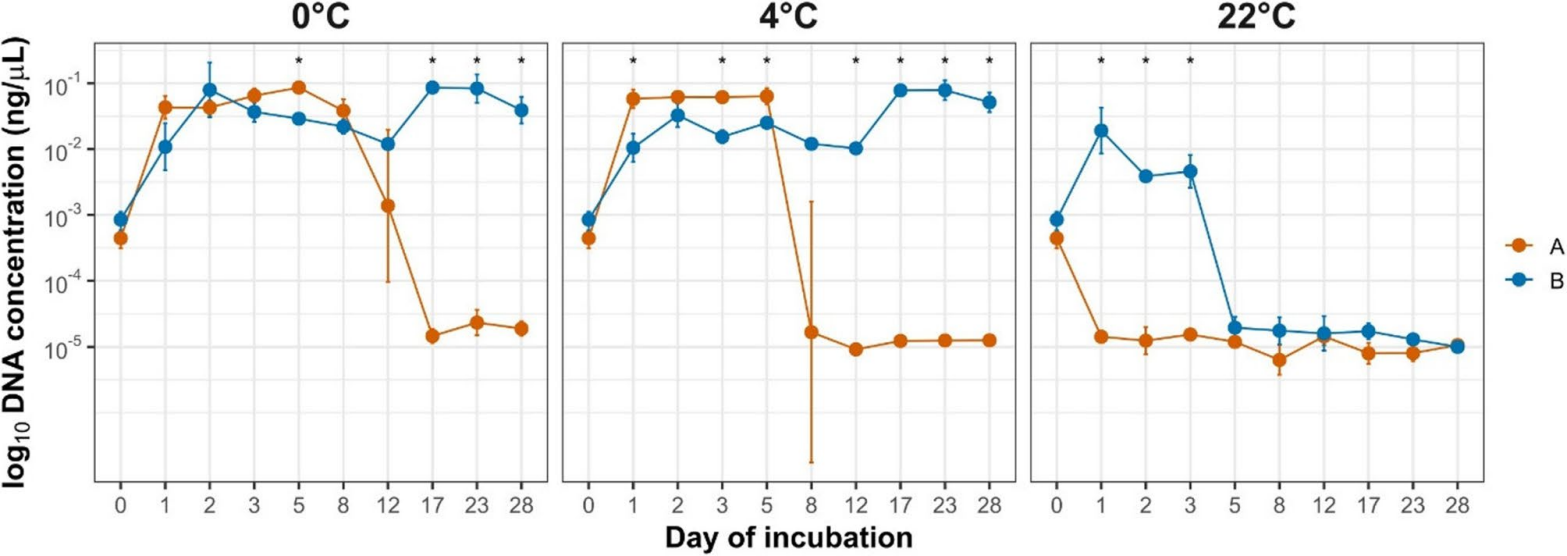
DNA concentration (log-transformed) in broth culture samples of *M. anserisalpingitidis* strains A and B over a 28-day incubation period at 0 °C, 4 °C, and 22 °C. Points represent median values with interquartile ranges (IQR). Asterisks indicate statistically significant differences between strains at individual time points (t-test, *P* < 0.05)

**Fig. 4:**
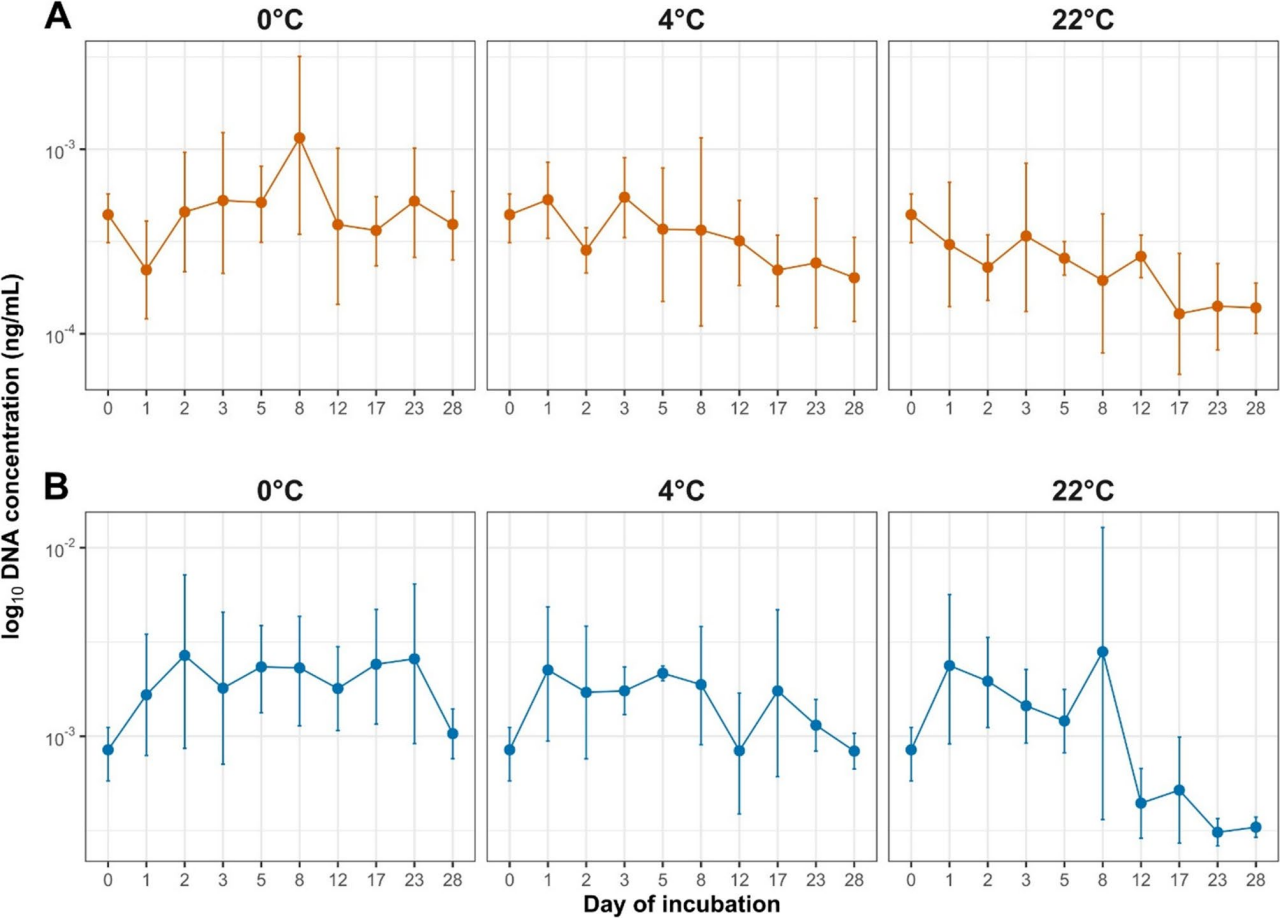
Concentration of *M. anserisalpingitidis* DNA in water over a 28-day incubation period at 0 °C, 4 °C, and 22 °C of strains (**A**) and (**B**). DNA concentration (ng/mL) was measured using qPCR and is presented on a log₁₀ scale. Data points represent median values with interquartile ranges (IQR)

The qPCR analysis was performed on broth cultures inoculated with water samples collected after incubation, providing information on DNA concentrations reflecting the bacterial population that remained viable and capable of growth under the tested temperature conditions (Fig. 3). For strain A, DNA concentration at 0 °C remained above the initial level until day 12 (0.00851 ng/µL), peaking on day 5 (0.08530 ng/µL), then dropping to 0.00001 ng/µL by day 17. At 4 °C, increased DNA concentration persisted until day 8 (0.02738 ng/µL). At 22 °C, no increase above the baseline was observed. For strain B, DNA concentration at 0 °C stayed above the baseline throughout the experiment, peaking on day 17 (0.08891 ng/µL). At 4 °C, elevated concentrations were maintained for the full duration, with the highest value observed on day 23 (0.09908 ng/µL). At 22 °C, DNA concentration remained above initial level until 3 days, with the highest value recorded on day 1 (0.03095 ng/µL). The qPCR data showed that strain B consistently maintained higher DNA concentrations after incubation, indicating that more bacteria retained the capacity to grow under lower temperature conditions. However, for both strains, fluctuations in DNA concentration were observed between individual time points (Fig. 4).

Our results showed that both strain identity and incubation temperature significantly influenced survival outcomes.

## Discussion

This study demonstrated that the survival of *M. anserisalpingitidis* in water is strongly influenced by both temperature and strain. Although both strains survived longer at lower temperatures, strain B demonstrated markedly greater persistence than strain A. At 0 °C, strain A remained viable for 12 days, whereas strain B retained viability throughout the 28-day experiment. A similar pattern was observed at 4 °C, with strain A viable for up to 8 days and strain B again persisting for the full duration. Even the relatively small difference between 0 °C and 4 °C led to a statistically significant reduction in viability for strain A. The prolonged survival observed under cold conditions is likely related to reduced metabolic activity, which may promote energy conservation and slow cellular degradation in both strains. In such environments, bacterial cells could benefit from nutrients released by lysed cells. In addition to nutrient recycling, bacteria may activate survival mechanisms such as autophagy and cannibalism under environmental stress [28]. Although water is generally considered an unfavorable medium for *Mycoplasma*, due to the lack of essential components such as cholesterol, the gradual degradation of non-viable cells might have provided limited but sufficient nutrients to support survival. This effect may have been especially important under low-temperature conditions, where cell lysis and degradation proceed more slowly, potentially extending resource availability.

Our findings are in agreement with previous studies that demonstrated a strong impact of environmental temperature on *Mycoplasma* survival [29]. In contrast to earlier reports for *M. gallisepticum* and *M. synoviae*, where survival in water did not exceed 7 days [4, 5], strain B in our study remained viable for at least 28 days. However, since the duration of the experiment was limited to 28 days, we were not able to determine the true upper limit of environmental persistence for *M. anserisalpingitidis*. Other members of the *Mycoplasma* genus have been shown to survive up to 8 months under farm conditions, with humidity playing a key role [30].

We used qPCR with a standard curve to quantify DNA concentrations in broth cultures inoculated with water samples collected after incubation, providing additional information on differences between the strains in their ability to recover and multiply after exposure to stress conditions. Strain B reached the highest DNA concentrations at 4 °C, while strain A peaked at 0 °C. This strain-dependent response is consistent with broader evidence of phenotypic diversity within bacterial species [31, 32]. One such mechanism could involve biofilm formation, a known survival strategy under suboptimal conditions [33]. Although biofilm formation was not assessed experimentally in this study, it may partially explain the prolonged survival observed in strain B. Biofilm formation is influenced by temperature and nutrient limitation. Studies on *Pseudomonas fluorescens* have shown that some isolates form biofilms more effectively at lower temperatures [34], while nutritional stress can also promote biofilm production [35]. In *M. anserisalpingitidis*, biofilm-producing strains demonstrate increased resistance to heat and desiccation, although this does not necessarily correlate with antibiotic resistance [33]. Studies on *M. gallisepticum* and *M. synoviae* [36] also indicate that biofilm formation may be a strain-specific trait.

Environmental conditions clearly influence the ability of mycoplasmas to survive outside the host. Starvation and temperature stress have profound effects on bacterial physiology. Under such conditions, bacteria progress through distinct growth phases, including long-term stationary phase, which can persist for extended periods [37]. During this phase, mutant variants better adapted to stress may gain a survival advantage [38, 39]. This could explain the fluctuating DNA concentrations observed in our study. In natural settings, bacteria often exist in a stationary-like physiological state that supports long-term survival under unfavorable conditions [11]. Studies on *M. gallisepticum* and *A. laidlawii* have demonstrated stress-induced ultrastructural changes, such as nanoform development and protein profile alteration [40–42]. These adaptations may involve shifts in transcriptional and proteomic responses, including expression of stress-related proteins like RNA polymerase β-subunit and Mar, which have been linked to antimicrobial resistance [43]. While these mechanisms were not investigated here, they remain important subjects for future research.

Although water is essential for animal health, it may also serve as a vector for infectious agents. Contaminants such as *Escherichia coli*, *Salmonella* spp., and *Campylobacter* spp. have been identified in poultry drinking water [44–46]. High microbial loads in water systems can negatively affect poultry performance [47]. Additionally, antibiotic residues [48] and resistance genes have been detected in water used in animal production [44].

In waterfowl farming systems, water sources such as troughs and natural bodies may act as reservoirs for *M. anserisalpingitidis*. These environments reduce the risk of desiccation and overheating but may expose bacteria to low temperatures.

Our results support the recognition of water and water-related infrastructure as potential environmental reservoirs of *M. anserisalpingitidis*. Moreover, the observed variation in survival between strains highlights that environmental persistence may not be uniform across isolates. While the present study included only two strains, most previous studies investigating environmental survival of avian mycoplasmas have relied on single-strain data. Our findings suggest that broader strain-level assessments are needed to more accurately estimate the risks associated with indirect transmission routes in waterfowl production systems.

As the minimal infective dose for *M. anserisalpingitidis* remains unknown, even low environmental concentrations may carry epidemiological relevance.

Taken together, these findings demonstrate that the environmental survival of *M. anserisalpingitidis* is both strain- and temperature-dependent, and emphasize the potential role of water sources as reservoirs contributing to indirect transmission. These results underline the importance of implementing effective water management and biosecurity measures in waterfowl production systems to minimize environmental exposure and reduce transmission risks.

## Conclusions

This study demonstrates that the survival of *M. anserisalpingitidis* in water is both temperature- and strain-dependent. Strain B remained viable for the entire 28-day experiment at 0 °C and 4 °C, while strain A exhibited significantly shorter survival at all tested temperatures. The results obtained by culture-based methods were supported by consistent DNA concentration patterns observed in broth cultures analyzed by qPCR. The findings highlight the potential role of water as a reservoir for *M. anserisalpingitidis*, and emphasize the need to consider strain-level variability when assessing transmission risks. Effective water management and biosecurity measures should be incorporated into control strategies in waterfowl production systems to minimize environmental exposure and reduce infection risks.

## Supplementary Information

Supplementary Material 1.

## Data Availability

Data generated or analysed during this study are included in this published article and its Supplementary dataset.

## References

[1] Ferguson-Noel N, Armour NK, Noormohammadi AH, El-Gazzar M, Bradbury JM. Mycoplasmosis. In: Swayne DE, editor. Diseases of Poultry. 14th ed. Hoboken, NJ, USA: John Wiley & Sons, Inc.; 2020. p. 907–65.

[2] Volokhov DV, Grózner D, Gyuranecz M, Ferguson-Noel N, Gao Y, Bradbury JM, et al. *Mycoplasma anserisalpingitidis* sp. *nov.*, isolated from European domestic geese (*Anser anser domesticus*) with reproductive pathology. Int J Syst Evol Microbiol. 2020;70:2369–81. 10.1099/ijsem.0.004052. 32068526 10.1099/ijsem.0.004052

[3] Sawicka-Durkalec A, Tomczyk G, Kursa O, Stenzel T, Gyuranecz M. Evidence of *Mycoplasma* spp. Transmission by migratory wild geese. Poult Sci. 2022;101:101526. 10.1016/j.psj.2021.101526. 34823180 10.1016/j.psj.2021.101526 PMC8627964

[4] Polak-Vogelzang AA. Survival of *Mycoplasma gallisepticum* in mains water. Avian Pathol. 1977;6:93–5. 10.1080/03079457708418215. 18770315 10.1080/03079457708418215

[5] Marois C, Savoye C, Kobisch M, Kempf I. A reverse transcription-PCR assay to detect viable *Mycoplasma synoviae* in poultry environmental samples. Vet Microbiol. 2002;89:17–28. 10.1016/S0378-1135(02)00159-1. 12223159 10.1016/s0378-1135(02)00159-1

[6] Marois C, Dufour-Gesbert F, Kempf I. Polymerase chain reaction for detection of *Mycoplasma gallisepticum* in environmental samples. Avian Pathol. 2002;31:163–8. 10.1080/03079450120118658. 12396361 10.1080/03079450120118658

[7] Marois C, Picault J-P, Kobisch M, Kempf I. Experimental evidence of indirect transmission of *Mycoplasma synoviae*. Vet Res. 2005;36:759–69. 10.1051/vetres:2005031. 16120251 10.1051/vetres:2005031

[8] Münster P, Kemper N. Long-term analysis of drinking water quality in poultry and pig farms in Northwest Germany. Front Anim Sci. 2024;5:1467287. 10.3389/fanim.2024.1467287.

[9] Elmberg J, Berg C, Lerner H, Waldenström J, Hessel R. Potential disease transmission from wild geese and swans to livestock, poultry and humans : a review of the scientific literature from a one health perspective. Infect Ecol Epidemiol. 2017;7. 10.1080/20008686.2017.1300450. 10.1080/20008686.2017.1300450 PMC5443079 28567210

[10] Abulreesh HH, Paget TA, Goulder R. Waterfowl and the bacteriological quality of amenity ponds. J Water Health. 2004;2:183–9. 10.2166/wh.2004.0016. 15497814

[11] Gonzalez JM, Aranda B. Microbial growth under limiting Conditions-Future perspectives. Microorganisms. 2023;11:1641. 10.3390/microorganisms11071641. 37512814 10.3390/microorganisms11071641 PMC10383181

[12] Arana I, Muela A, Orruño M, Seco C, Garaizabal I, Barcina I. Effect of temperature and starvation upon survival strategies of *Pseudomonas fluorescens* CHA0: comparison with *Escherichia coli*. FEMS Microbiol Ecol. 2010;74:500–9. 10.1111/j.1574-6941.2010.00979.x. 20955194 10.1111/j.1574-6941.2010.00979.x

[13] Nedwell DB. Effect of low temperature on microbial growth: Lowered affinity for substrates limits growth at low temperature. FEMS Microbiol Ecol. 2006;30:101–11. 10.1111/j.1574-6941.1999.tb00639.x. 10.1111/j.1574-6941.1999.tb00639.x 10508935

[14] Marmion M, Macori G, Ferone M, Whyte P, Scannell AGM. Survive and thrive: control mechanisms that facilitate bacterial adaptation to survive manufacturing-related stress. Int J Food Microbiol. 2022;368:109612. 10.1016/j.ijfoodmicro.2022.109612. 35278797 10.1016/j.ijfoodmicro.2022.109612

[15] Moon S, Ham S, Jeong J, Ku H, Kim H, Lee C. Temperature matters: bacterial response to temperature change. J Microbiol. 2023;61:343–57. 10.1007/s12275-023-00031-x. 37010795 10.1007/s12275-023-00031-x

[16] Citti C, Blanchard A. Mycoplasmas and their host: emerging and re-emerging minimal pathogens. Trends Microbiol. 2013;21:196–203. 10.1016/j.tim.2013.01.003. 23419218 10.1016/j.tim.2013.01.003

[17] Rocha EPC, Blanchard A. Genomic repeats, genome plasticity and the dynamics of *Mycoplasma* evolution. Nucleic Acids Res. 2002;30:2031–42. 10.1093/nar/30.9.2031. 11972343 10.1093/nar/30.9.2031 PMC113839

[18] Katz SD. The Streak Plate Protocol. Am Soc Microbiol. 2008;1–10. https://asm.org/asm/media/protocol-images/the-streak-plate-protocol.pdf.

[19] Grózner D, Sulyok KM, Kreizinger Z, Rónai Z, Jánosi S, Turcsányi I, et al. Detection of *Mycoplasma anatis*, *M. anseris*, *M. cloacale* and *Mycoplasma* sp. 1220 in waterfowl using species-specific PCR assays. PLoS ONE. 2019;14:e0219071. 10.1371/journal.pone.0219071. 31295269 10.1371/journal.pone.0219071 PMC6622482

[20] Gioia G, Werner B, Nydam DV, Moroni P. Validation of a Mycoplasma molecular diagnostic test and distribution of Mycoplasma species in bovine milk among new York state dairy farms. J Dairy Sci. 2016;99:4668–77. 10.3168/jds.2015-10724. 27016831 10.3168/jds.2015-10724

[21] Hannan PCT. Guidelines and recommendations for antimicrobial minimum inhibitory concentration (MIC) testing against veterinary Mycoplasma species. Vet Res. 2000;31:373–95. 10.1051/vetres:2000100. 10958240 10.1051/vetres:2000100

[22] Bekő K, Grózner D, Mitter A, Udvari L, Földi D, Wehmann E, et al. Development and evaluation of temperature-sensitive *Mycoplasma anserisalpingitidis* clones as vaccine candidates. Avian Pathol. 2022;51:535–49. 10.1080/03079457.2022.2102967. 35866306 10.1080/03079457.2022.2102967

[23] Terry M. Therneau, Patricia M. Grambsch. Modeling Survival Data: Extending the Cox Model. New York: Springer; 2000.

[24] Tang Y, Horikoshi M, Li W. Ggfortify: unified interface to visualize statistical results of popular R packages. R J. 2016;8:474. 10.32614/RJ-2016-060.

[25] Wickham H. ggplot2: Elegant Graphics for Data Analysis. New York: Springer-Verlag; 2016. 10.1007/978-3-319-24277-4.

[26] R Core Team. R: A Language and environment for statistical computing. Vienna, Austria: Foundation for Statistical Computing; 2025.

[27] Posit team. RStudio: Integrated Development Environment for R. Posit Software, PBC, Boston: MA; 2025. http://www.posit.co/.

[28] Merchant SS, Helmann JD. Elemental economy: microbial strategies for optimizing growth in the face of nutrient limitation. Adv Microb Physiol. 2012;91–210. 10.1016/B978-0-12-398264-3.00002-4. 10.1016/B978-0-12-398264-3.00002-4 PMC4100946 22633059

[29] Nagatomo H. Comparative studies of the persistence of animal Mycoplasmas under different environmental conditions. Vet Microbiol. 2001;82:223–32. 10.1016/S0378-1135(01)00385-6. 11470544 10.1016/s0378-1135(01)00385-6

[30] Justice-Allen A, Trujillo J, Corbett R, Harding R, Goodell G, Wilson D. Survival and replication of *Mycoplasma* species in recycled bedding sand and association with mastitis on dairy farms in Utah. J Dairy Sci. 2010;93:192–202. 10.3168/jds.2009-2474. 20059918 10.3168/jds.2009-2474

[31] Nouvel LX, Sirand-Pugnet P, Marenda MS, Sagné E, Barbe V, Mangenot S, et al. Comparative genomic and proteomic analyses of two *Mycoplasma agalactiae* strains: clues to the macro- and micro-events that are shaping Mycoplasma diversity. BMC Genomics. 2010;11:86. 10.1186/1471-2164-11-86. 20122262 10.1186/1471-2164-11-86 PMC2824730

[32] Delaney NF, Balenger S, Bonneaud C, Marx CJ, Hill GE, Ferguson-Noel N, et al. Ultrafast evolution and loss of crisprs following a host shift in a novel wildlife pathogen, *Mycoplasma gallisepticum*. PLoS Genet. 2012;8:e1002511. 10.1371/journal.pgen.1002511. 22346765 10.1371/journal.pgen.1002511 PMC3276549

[33] Bekő K, Nagy EZ, Grózner D, Kreizinger Z, Gyuranecz M. Biofilm formation and its impact on environmental survival and antibiotic resistance of *Mycoplasma anserisalpingitidis* strains. Acta Vet Hung. 2022;70:184–91. 10.1556/004.2022.00029. 36178765 10.1556/004.2022.00029

[34] Rossi C, Chaves-López C, Serio A, Goffredo E, Cenci Goga BT, Paparella A. Influence of incubation conditions on biofilm formation by Pseudomonas fluorescens isolated from dairy products and dairy manufacturing plants. Ital J Food Saf. 2016;5. 10.4081/ijfs.2016.5793. 10.4081/ijfs.2016.5793 PMC5090116 27853712

[35] De Plano LM, Caratozzolo M, Conoci S, Guglielmino SPP, Franco D. Impact of nutrient starvation on biofilm formation in *Pseudomonas aeruginosa*: an analysis of growth, adhesion, and Spatial distribution. Antibiotics. 2024;13:987. 10.3390/antibiotics13100987. 39452253 10.3390/antibiotics13100987 PMC11504098

[36] Catania S, Bottinelli M, Fincato A, Tondo A, Matucci A, Nai G, et al. Pathogenic avian Mycoplasmas show phenotypic differences in their biofilm forming ability compared to non-pathogenic species in vitro. Biofilm. 2024;7:100190. 10.1016/j.bioflm.2024.100190. 38515541 10.1016/j.bioflm.2024.100190 PMC10955283

[37] Pletnev P, Osterman I, Sergiev P, Bogdanov A, Dontsova O. Survival guide: *Escherichia coli* in the stationary phase. Acta Naturae. 2015;7:22–33. 26798489 PMC4717247

[38] Navarro Llorens JM, Tormo A, Martínez-García E. Stationary phase in gram-negative bacteria. FEMS Microbiol Rev. 2010;34:476–95. 10.1111/j.1574-6976.2010.00213.x. 20236330 10.1111/j.1574-6976.2010.00213.x

[39] Hazan R, Schoemann M, Klutstein M. Endurance of extremely prolonged nutrient prevention across kingdoms of life. iScience. 2021;24:102745. 10.1016/j.isci. 34258566 10.1016/j.isci.2021.102745 PMC8258982

[40] Chernov VM, Gogolev YV, Mukhametshina NE, Abdrakhimov FA, Chernova OA. Mycoplasma adaptation to biogenic and abiogenic stessful factors; *Acholeplasma laidlawii* nannotransformation and minibodies. Prog Nucl Energy 6 Biol Sci. 2003;396:417–20. 10.0012/4966/04/0506-0251. 10.1023/b:dobs.0000033291.33157.a2 15354840

[41] Demina IA, Serebryakova MV, Ladygina VG, Rogova MA, Kondratov IG, Renteeva AN, et al. Proteomic characterization of *Mycoplasma gallisepticum* nanoforming. Biochem (Moscow). 2010;75:1252–7. 10.1134/S0006297910100068. 10.1134/s0006297910100068 21166642

[42] Chernov VM, Chernova OA, Gorshkov OV, Muzykantov AA, Shaimardanova GF, Pel’nikevich AD, et al. Adaptation of *Mycoplasma gallisepticum* to unfavorable growth conditions: changes in morphological and physiological characteristics. Microbiol (N Y). 2008;77:691–4. 10.1134/S0026261708060064. 19137716

[43] Chernov VM, Chernova OA, Medvedeva ES, Sorvina AI, Davydova MN, Rogova MA, et al. Responses of *Acholeplasma Laidlawii* PG8 cells to cold shock and oxidative stress: proteomic analysis and stress-reactive Mycoplasma proteins. Dokl Biochem Biophys. 2010;432:126–30. 10.1134/S1607672910030099. 20886746 10.1134/s1607672910030099

[44] Piccirillo A, Tolosi R, Mughini-Gras L, Kers JG, Laconi A. Drinking water and biofilm as sources of antimicrobial resistance in Free-Range organic broiler farms. Antibiotics. 2024;13:808. 10.3390/antibiotics13090808. 39334983 10.3390/antibiotics13090808 PMC11429059

[45] Mustedanagic A, Matt M, Weyermair K, Schrattenecker A, Kubitza I, Firth CL, et al. Assessment of microbial quality in poultry drinking water on farms in Austria. Front Vet Sci. 2023;10:1254442. 10.3389/fvets.2023.1254442. 38076551 10.3389/fvets.2023.1254442 PMC10702765

[46] Kapperud G, Skjerve E, Vik L, Hauge K, Lysaker A, Aalmen I, et al. Epidemiological investigation of risk factors for Campylobacter colonization in Norwegian broiler flocks. Epidemiol Infect. 1993;111:245–55. 10.1017/s0950268800056958. 8405152 10.1017/s0950268800056958 PMC2271384

[47] Sparks NHC. The role of the water supply system in the infection and control of Campylobacter in chicken. Worlds Poult Sci J. 2009;65:459–74. 10.1017/S0043933909000324.

[48] Gbylik-Sikorska M, Posyniak A, Sniegocki T, Sell B, Gajda A, Sawicka A, et al. Influence of enrofloxacin traces in drinking water to doxycycline tissue pharmacokinetics in healthy and infected by Mycoplasma gallisepticum broiler chickens. Food Chem Toxicol. 2016;90:123–9. 10.1016/j.fct.2016.02.006. 26875641 10.1016/j.fct.2016.02.006

